# Acute lower respiratory infections among children under five in Sub-Saharan Africa: a scoping review of prevalence and risk factors

**DOI:** 10.1186/s12887-023-04033-x

**Published:** 2023-05-06

**Authors:** Jacob Owusu Sarfo, Mustapha Amoadu, Thomas Boateng Gyan, Abdul-Ganiyu Osman, Peace Yaa Kordorwu, Abdul Karim Adams, Immanuel Asiedu, Edward Wilson Ansah, Forster Amponsah-Manu, Priscilla Ofosu-Appiah

**Affiliations:** 1 Department of Health, Physical Education and Recreation, University of Cape Coast, Cape Coast, Ghana; 2 Eastern Regional Hospital, Koforidua, Ghana

**Keywords:** Acute lower respiratory infection, Children under five, Sub-Saharan Africa

## Abstract

**Background:**

Acute lower respiratory tract infections (ALRTIs) among children under five are still the leading cause of mortality among this group of children in low and middle-income countries (LMICs), especially countries in sub-Saharan Africa (SSA). This scoping review aims to map evidence on prevalence and risk factors associated with ALRTIs among children under 5 years to inform interventions, policies and future studies.

**Methods:**

A thorough search was conducted via four main databases (PubMed, JSTOR, Web of Science and Central). In all, 3,329 records were identified, and 107 full-text studies were considered for evaluation after vigorous screening and removing duplicates, of which 43 were included in this scoping review.

**Findings:**

Findings indicate a high prevalence (between 1.9% to 60.2%) of ALRTIs among children under five in SSA. Poor education, poverty, malnutrition, exposure to second-hand smoke, poor ventilation, HIV, traditional cooking stoves, unclean fuel usage, poor sanitation facilities and unclean drinking water make children under five more vulnerable to ALRTIs in SSA. Also, health promotion strategies like health education have doubled the health-seeking behaviours of mothers of children under 5 years against ALRTIs.

**Conclusion:**

ALRTIs among children under five still present a significant disease burden in SSA. Therefore, there is a need for intersectoral collaboration to reduce the burden of ALRTIs among children under five by strengthening poverty alleviation strategies, improving living conditions, optimising child nutrition, and ensuring that all children have access to clean water. There is also the need for high-quality studies where confounding variables in ALRTIs are controlled.

## Introduction

Acute lower respiratory tract infections (ALRTIs) are infections in the trachea, lungs, bronchi, bronchioles, and alveoli [1]. Common ALRTIs usually include pneumonia, bronchitis and bronchiolitis [1]. ALRTIs are the leading cause of diseases and deaths among children under 5 years globally [2, 3]. It is worth noting that about 97% of all cases of ALRTIs are reported in low-and middle-income countries (LMICs), with most cases (about 70%) coming from South Asia and sub-Saharan Africa [SSA] [4]. Pneumonia kills more children than any other infectious disease, accounting for over 800,000 mortalities in children under 5 years annually [4]. Thus, each day about 2,200 children under five die of pneumonia. The highest deaths come from South Asia (2,500 cases per 100,000 children under five) and SSA (1,620 cases per 100,000 children under five). Unfortunately, all these deaths could have been prevented [1]. Data from UNICEF shows that progress in reducing pneumonia deaths in children under five has been significantly slower than in other infectious diseases such as diarrhoea, sepsis and malaria, especially in resource-poor settings like SSA [4].

The pathogens that cause ALRTIs vary depending on the person’s age [5]. *Streptococcus pyogenes*, *Pneumococci*, *Staphylococcus aureus*, *Klebsiella pneumonia*, and *Haemophilus influenzae* are the known causes of bacterial ALRTIs among children under 5 years of age [5, 6]. In addition, respiratory syncytial virus (RSV), parainfluenza type 3 virus (PF3), adenovirus (Adeno), influenza virus (FLU), and enterovirus are the common viruses that cause ALRTIs in children under 5 years of age [6]. Antibiotics are effective against most bacterial infections. However, evidence shows that diversity of the organisms causing ALRTIs and the deficit (about 30%) in diagnosis makes its management and treatment among children challenging [6]. Perhaps, these treatment challenges might be the reason for slower than expected reduction in ALRTIs among under five [4]. Hence, one of the best ways of achieving a significant reduction in morbidities and mortalities associated with ALRTIs among under five children is prevention of the disease through disruption of transmission of pathogens and reduction in risk factors.

The evidence further shows that several factors are linked to ALRTIs among children under 5 years [7]. However, Seidu and colleagues argued that there might be variations in these factors in LMICS and high-income countries [6]. This means that effective policies and strategies developed based on risk factors in high-resourced countries for preventing ALRTIs among children under five might not be effective in preventing the same among population in low-resourced countries, especially countries in SSA. Hence, it is important to identify the risk factors for ALRTIs, which are pertinent for developing effective policies and interventions to interrupt the transmission of ALRTIs pathogens and to ensure improved health outcomes.

For decades of research into ALRTIs, reviews mapping the prevalence, risk factors and interventions for ALRTIs among children under 5 years in SSA are scarce.. Some reviews considered pathogens [8] and air pollution as risk factors for ALRTIs among children under 5 years [9]. Therefore, this scoping review aims to map evidence on the prevalence and risk factors of ALRTIs among children under 5 years in SSA to inform future studies and help develop robust and effective policies and interventions in the prevention and protection of children under 5 years from infection. Perhaps, prevention becomes the best and most cost-effective strategy for protecting children under 5 years of age from ALRTIs since countries in SSA are already facing challenges in health systems, diagnosis and treatment of ALRTIs [6, 8, 10].

## Methods

This scoping review adopted the guidelines of Arksey and O’Malley [11]. We also adopted the Preferred Reporting Items for Systematic Reviews and Meta-Analyses extension for Scoping Reviews (PRISMA-ScR) checklist [12]. Three research questions guided this review: (1) what is the prevalence of reported ALRTIs among children under 5 years in SSA? (2) what are the risk factors of ALRTIS among children under 5 years in SSA? and (3) what interventions serve as protective factors for children under five in SSA against ALRTIs?

To get relevant studies for this review, the authors developed eligibility criteria. Table 1 presents the search strategy and the eligibility criteria. The search was conducted in four main databases (PubMed, JSTOR, Central, and Web of Science). An initial search was conducted in PubMed using Medical Subject Headings (MeSH) and later adapted to other databases. A chartered librarian, Dr Kwame Kodua-Ntim, at the Sam Jonah Library was consulted for records search and data management. Additional sources such as The WHO Library, Maternal Surveillance and Response Action Network, Google Scholar, Google, Z-library, HINARI, and institutional repositories of some universities in SSA were searched for relevant records. Mendeley software was used to remove duplicates and manage records during the screening process. All authors were involved in the screening for relevant records for this review. The reference lists of eligible studies were checked to identify relevant papers for this review.

**Table 1:** Search strategy for articles on ALRTIs among children under five in SSA

**Search strategy item**	
Databases	PubMed, JSTOR, Central, Web of Science
Language filter	English Language
Time filter	2010 -2022
Spatial filter	Sub-Saharan African Countries Angola OR Benin OR Botswana OR Burkina Faso OR Burundi OR Cape Verde OR Cameroon OR Central African republic OR Chad OR Comoros OR Congo OR DR, Congo OR Coted’Ivoire OR Equatorial Guinea OR Eritrea OR Eswatini OR Ethiopia OR Gabon OR Gambia Ghana OR Guinea OR Guinea Bissau OR Kenya OR Lesotho OR Liberia OR Madagascar OR Malawi OR Mali OR Mauritania OR Mauritius OR Mozambique OR Namibia OR Niger OR Nigeria OR Rwanda OR Sao Tome & Principe OR Senegal OR Seychelles OR Sierra Leon OR Somalia OR South Africa OR South Sudan OR Sudan OR Tanzania OR Togo OR Uganda OR Zambia OR Zimbabwe
Keywords	1. Acute Lower Respiratory Infection OR Respiratory Infection OR Lower Respiratory Tract Infection OR Pneumonia OR Influenza OR Whooping Cough OR Acute Bronchitis OR Bronchiolitis 2. Children under Five Years OR Infants OR Neonates OR Early Childhood OR Children 3. Prevalence OR Percentage of Diseases or Proportion 4. Risk Factors OR Causes OR Determinants OR Exposure 5. Interventions OR Policies or Strategies OR Protection
Inclusion criteria	The paper should be: 1. A peer-reviewed or Gray Literature; 2. published from 2010 to 2022; 3. conducted in sub-Saharan African countries; 4. published in the English language; 5. conducted on children under five years, 6. on Prevalence, Risk Factors or Interventions, any of these three outcomes
Exclusion criteria	The paper should be 1. reviews, reports, abstracts, minutes, commentaries, letters to editors, and preprints 2. conducted on children above five years, 3. conducted in countries outside sub-Saharan Africa; 4. studies published online before the year 2010

To ensure accuracy and consistency in our approach to this review, two groups of authors (TBG, A-GO, and PYK; AKA and IA) independently extracted the data. Discrepancies during data charting were resolved during regular meetings by all the authors. The data charting process was supervised and reviewed by JOS and MA. In addition, EWA (Health Promotion expert), FA-M (Medical expert and Consultant General Surgeon), and PO-A (Paediatric Nurse Specialist) served as independent subjects and review experts that guided the entire review process. The analysis involved thematic and content analysis.

## Results

**Fig. 1:**
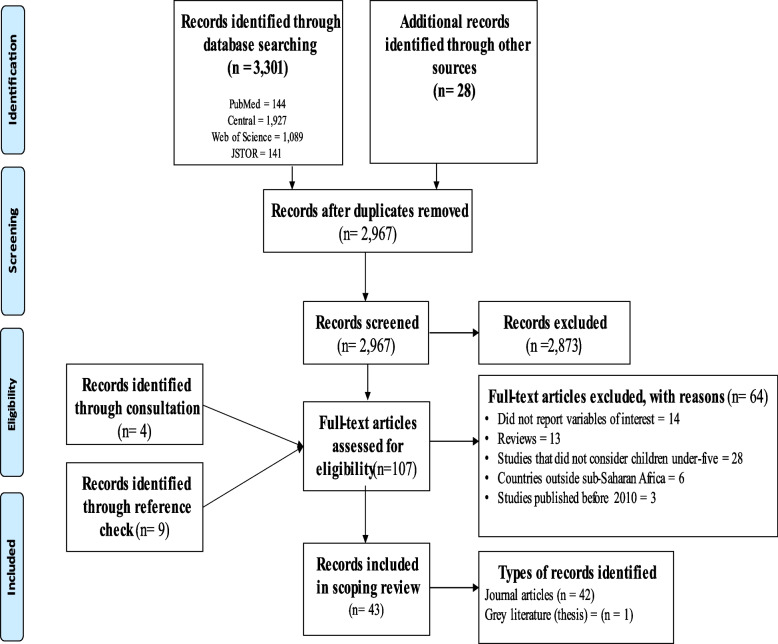
PRISMA flow diagram of articles on ALRTIs among children under five in SSA

The search was done in the main four databases (PubMed, Central, Web of Science and JSTOR) and produced 3,301 records. In addition, 28 records were ascertained from searches in other sources such as google, Z-library.org, institutional repositories, and google scholar. After the removal of 362 duplicates, 2,967 records were further screened. Finally, 94 full-text records were assessed for eligibility. Consultations and reference checking produced additional 13 full-text records for eligibility assessment. Of the 107 full-text records, 43 were finally included in this scoping review. Details of the search results and screening process are presented in Fig. 1.

### Characteristics of included studies

**Fig. 2:**
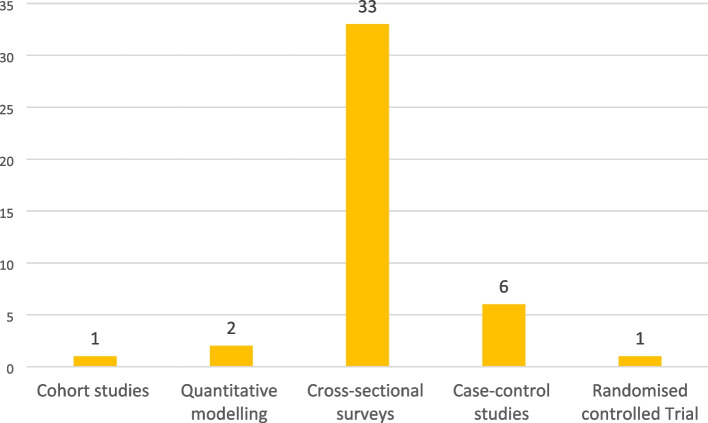
Study designs of included studies on ALRTIs among children under five in SSA

**Fig. 3:**
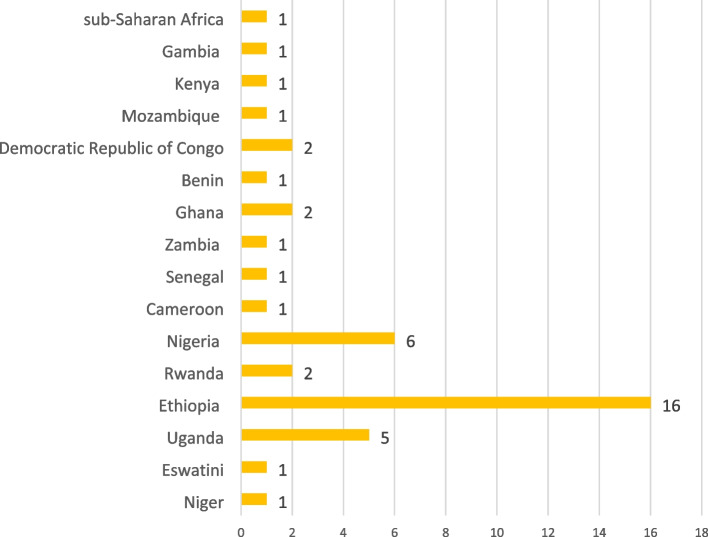
Countries included studies on ALRTIs among children under five in SSA

**Fig. 4:**
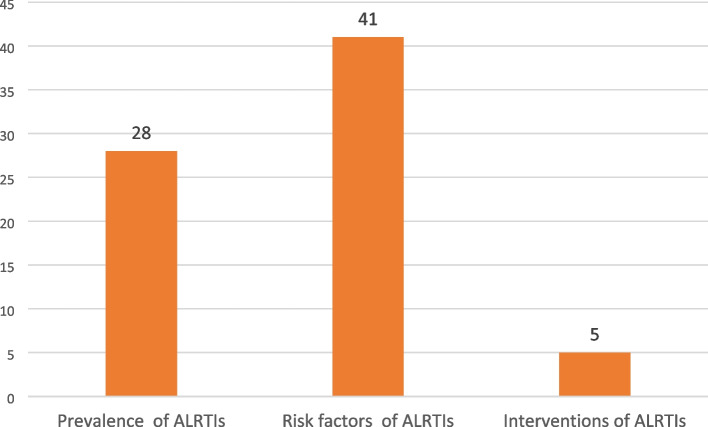
Studies based on objectives of the review on ALRTIs among children under five in SSA

The majority of the included studies were cross-sectional surveys (33) and case–control studies (6). See the details of the study designs of included studies in Fig. 2. Most of the included studies were conducted in Ethiopia (16), Nigeria (6) and Uganda (5). See the details of countries where included studies were conducted in Fig. 3. Included studies used a total sample size of 526,667. Furthermore, most of the reviewed studies were conducted on risk factors (41) and prevalence (28). See the details of reviewed studies based on the objectives of this scoping review in Fig. 4. Also, the details of extracted data are presented in Table 2.

**Table 2:** Data extraction for included studies

Author and country	Purpose of the study	Design	Population	Sample size	Prevalence	Risk factors	Intervention/ policies
[1] Ethiopia	To assess the magnitude of lower respiratory tract infections and associated factors among under five children visiting Wolaita Sodo University Teaching and Referral Hospital	Cross-sectional survey	Mothers and care-takers (Hospitalised sample)	414	40.3%	Unvaccinated children, non-exclusive breastfeeding or replacement breastfeeding, unclean fuel for cooking, absence of separate kitchen, absence of window in the kitchen room	
[13] Dr. Congo	To evaluate viral co-infections and risk factors for lower respiratory tract infections in children under 5 years	cross-sectional study	Children under five – years (Hospitalised sample)	146	57.5%	Malnutrition, rural settings, low income and mother illiteracy were risk factors	
[14] Nigeria	To evaluate risk Factors for Acute Respiratory Tract Infections in Under‑five Children	cross-sectional study	Children Under – 5 Years (Hospitalised sample)	436	Pneumonia (31.6%), Bronchiolitis (6.9%)	Undernourished, Inadequate breast feeding, poor immunisation, attendance to day care centres, large family size, poor parental educational status, parental smoking, living in urban areas and the use of biofuels were risk factors	
[15] Cameroon	To determine the proportion of acute respiratory infections and the associated risk factors in children under 5 years visiting the Bamenda Regional Hospital in Cameroon	Cross-sectional survey	Children under five (Hospitalised sample)	512	Pneumonia (22.3%)	HIV infection, poor maternal education, exposure to wood smoke, passive smoking and contact with someone who has cough	
[16] Ethiopia	To evaluate the prevalence of, and risk factors associated with, acute respiratory infection hospitalisation in under five years children hospitalised at the University of Gondar Comprehensive Specialized Hospital	Cross-sectional survey	Under five children (Hospitalised sample)	422	27.3%	Children < 2 years, younger maternal age, maternal age above 28 years, lack of awareness about handwashing, rural residence	
[17] Mozambique	To examine respiratory syncytial and influenza viruses in children under 2 years	Cross-sectional survey	Under two-years (Hospitalised sample)	450	26.7		
[18] Ethiopia	To evaluate association of acute respiratory infections with indoor air pollution from biomass fuel exposure among under five children	Cross sectional study	Under – 5 years (Community sample)	265	Pneumonia (13.2%), Bronchiolitis (1.9%)	Large family size and living in household with no separate kitchen	
[19] Rwanda	To examine spatial inequalities and Socioeconomic Factors of Acute Respiratory Infections among Under five Children	Cross sectional study	Under – 5 Years (community sample)	7,311	11.6%	Children with a history of diarrhoea, children > 2 years and crowded homes	
[20] Ethiopia	To evaluate respiratory symptoms and associated risk factors among under five children	Cross sectional survey	Under 5 Years (Community sample)	792	37.5%	Uterine irritability during pregnancy, physical exercise during pregnancy, using wood and coal for heating, cockroach infestation, presence of new carpets, damp stain, opening windows during cooking, living less than 100 m heavy traffic and living less than 100 m unpaved road or street are risk factors	
[21] Kenya	To examine long-term PM2.5 exposure as associated with symptoms of acute respiratory infections among children under five years of age	Cross sectional survey	Children under 5 Years (community sample)	7,036		Exposure to high concentrations of PM_2.5_ is a risk factor	
[22] Zambia	To examine the trends and factors associated with respiratory tract infection in children under five years	cross-sectional study	Under-5 years (Community sample)	30,391	5%	Underweight children, and the use of charcoal and firewood was associated with high ALRTIs, children of mothers with no education, breastfeeding children and crowded homes were risk factors. Mothers < 20 years	
[23] Ghana	To examine ecological zone and symptoms of acute respiratory infection among children under five	cross-sectional study	under five – years (Community sample)	3,393	22.0%	Mothers in rural areas	
[24] Ghana	To examine urinary pesticide residual levels and acute respiratory infections in children under 5 years of age	cross-sectional study	Mothers/ caretakers and children under five (Community sample)	404	22.1%	The use of agro-chemicals is a risk factor	
[25] Rwanda	To assess social, economic, and environmental factors associated with acute lower respiratory infections among children under five to inform potential further improvements in the health system	Cross-sectional study	Children under five (Community sample)	8,484		Children < 2 years, children with severe anaemia, children living in urban area and those who did not receive vitamin A, raining season	
[26] Ethiopia	To examine spatial distribution and determinants of acute respiratory infection among under five children	cross-sectional study	Children Under five- years **(**Community sample)	10,006		History of diarrhoea, children > 3 years, working mothers and stunting were risk factors	
[27] Uganda	To investigate the association between wood and charcoal domestic cooking, respiratory symptoms and acute respiratory infections among children aged under 5 years	Cross-Sectional study	Pre-school children (Community sample)	15,405		Wood fuel use	
[28] Ethiopia	To assess the prevalence and association factors of pneumonia among children under five in peri-urban area	cross-sectional study	Under five – years (Community sample)	560	17.1%	Cooking in the living room, overcrowding, malnutrition and child and family history of ALRTIs	
[6] Sub-Saharan Africa	To examine the prevalence and determinants of ALRIs among children under five years	Cross-sectional survey	Under-5 years (Community sample)	13,495	25.3%	Children aged 24–59 months and those who infected with intestinal parasite were at higher risk of ALRIs. Mothers who were employed and improved toilet facilities were protective factors	
[29] Ethiopia	To determine the risk factors for acute respiratory infection among children under the age of five in Ethiopia	cross-sectional survey	Children under five (Community sample)	9,918	8.4%	Children with mothers with low education, not receiving vitamin A, history diarrhoea and unimproved drinking water	
[30]	To determine the risk factors for acute respiratory infection in children under the age of five in rural Ethiopia	Cross-sectional survey	Children under five (Community sample)	7,911	7.8%	Poor household, mothers no education, child has not received vitamin A, child with history diarrhoea, mothers not working, stunted and no improved water source	
[31] Eswatini	To investigate the individual- and community-level factors associated with child ALRIs in Eswatini	Cross-sectional survey	Children under five (Community sample)	4,265	11.1%	Child born to women with no formal or primary education; history of diarrhoea; children from urban areas; children in households with low proportion of electricity	
[32] Ethiopia	To assess the association of food cooking place with acute respiratory infections and the variability in households and surveys	Cross-sectional survey	Children under -five (Community sample)	30,895	11.9%	Cooking inside the house, solid biomass fuel, breastfeeding, low parental education, mothers who do not listen or watch television and low wealth	
[33] Ethiopia	To investigate the spatiotemporal pattern of ALRI in Ethiopian administrative zones	Cross-sectional survey	Children under five (Community sample)	29,599	15%	Older children > 2, no formal parental education, children from poorer households, the use of unimproved water and toilet facilities, unclean fuel for cooking, underweight, stunted, history of diarrhoea	
[34] Ethiopia	To investigate the prevalence of childhood acute respiratory infection and associated factors in Northwest Ethiopia	Cross-sectional survey	Children under-four (Community sample)	5,830	19.2%	Living in house with no chimney, eaves space, improved cookstove, cow dung fuel, child spending time near stove, indoor cooking events, frequent cooking of meals	
[35] Ethiopia	To evaluate the association of biomass fuel use with acute respiratory infection in children under five years	Cross-sectional survey	Under-5 years (Community sample)	422	23.9%	Biomass, kerosine, Cigarette smoking, children who were held by their mothers while cooking, poor ventilation. Children who lived in male dominated home were less likely to be exposed to ALRTIs	
[36] Gambia	To examine prevalence and determinants of acute respiratory infections among children under five years	Cross-sectional survey	Rural mothers with children Under 5 Years (Community sample)	1364	37.1%	Children with mothers with primary education, non-breastfed children and children whose fathers were unemployed	
[37] Uganda	To analyse the risk factors of ARI disease symptoms among children under the age of 5 years in Uganda	Cross-sectional survey	Children under five (community sample)	13,493		Children < 2 years, one year child old, children whose mothers are teenagers and farm workers	
[38] Nigeria	To examine whether lifestyle factors are associated with ARI risk among Nigerian children aged less than 5 years, taking individual-level and contextual-level risk factors into consideration	Cross-sectional survey	Children under five (community sample)	28,596		In-house biomass cooking, no hand washing, orphan or vulnerable children	
[39] Nigeria	To investigate the specific regional determinants of overall and wealth-related inequality in children having ARI in Nigeria over a decade	Cross-sectional survey	Children under five (community sample)			No maternal education, having no vaccination card, having high birth order, short birth interval	
[40] Senegal	To explore this association by using the satellite-detected tropospheric NO2 concentrations measured by Sentinel-5 Precursor and ARI symptoms in children under age five	Cross-sectional survey	Children under five (community sample)	4,220		High level of nitrogen dioxide	
[41] Uganda	To evaluate household management of acute respiratory infections in children under five years	cross-sectional survey (Intervention study)	Mothers and caretakers of under five – years children (community sample)	200	Pneumonia (9%)	Appropriate use of medication is associated with decreased risk of ALRTIs, pneumonia symptoms and high level of education of caretaker	The management of ARIs among the under fives in Kampala is suboptimal with misuse of antibiotics, antimalarials, dexamethasone, herbal medicines and cough remedies common
[42] Uganda	To evaluate use of antibacterial in the management of symptoms of acute respiratory tract infections among children under five years	cross-sectional survey (Intervention study)	Children under five- years	865	60.2% prevalence of antibiotic usage	Getting treatment from a health facility, peri-urban area and child having cough	It was found that antibacterial use is more common in children who are taken to a health facility with symptoms of ARIs. We also observed that living in less remote (peri-urban) areas was associated with high frequency of antibacterial use compared to rural areas
[43] Uganda	To assess the secular trend in the prevalence of ARIs as well as their treatment seeking-behaviour among Ugandan infants	Cross-sectional survey (Intervention study)	Singleton infants 0–5 (Community sample)	26,974	32.7%	Children 2–5 years, high order birth, malnutrition, poor households, intendedness of the child; rural residency,	Treatment seeking behaviour has doubled since 1995
[44] Dr. Congo	To evaluate decreased number of hospitalised children with severe acute lower respiratory infection after introduction of the pneumococcal conjugate vaccine	Quantitative (modelling)	children under 5 Years (Hospitalised sample)	21,478	9.4%	Malnutrition	
[45] Benin	To examine high acute lower respiratory infection levels in children under five linked to specific weather conditions	Quantitative (modelling)	Under – 5 years	232,214		High relative humidity in wet season, low relative humidity and low temperature during dry season and high temperature	
[46] Niger	To improve the integrated management of childhood illness through identification of etiologies of respiratory infections for an adapted treatment and testing of a new strategy for post hospitalisation health monitoring	Cohort study (Intervention study)	Children under five (Hospitalised sample)	767	Pneumonia (59.2%)	Failure to receive the second dose of pentavalent vaccine	Multiple home visits for post hospitalisation health monitoring did not offer better prevention of morbidity and mortality compared to a single visit
[47] Ethiopia	To investigate the child health effect of improved baking stove intervention compared with the continuation of the open burning traditional baking stove	R.C.T (Intervention Study)	Children under five years (Community sample)	5,508	19.1%	Children > 2 years, inhouse cooking, frequency of cooking	Improved baking stove intervention did not have significant effect on ALRTI
[48] Ethiopia	To evaluate determinant factors for ARI	Case control	Under-5 years (hospitalised sample)	417		Age of the mother/ caregiver > 35 years, housewife, unclean stove, carrying the child while preparing food, absence of windows in the house, and nutritional status of the child	
[49] Nigeria	To examine the pattern of acute respiratory infections in hospitalised children under five	Case control study	Children Under – 5 years (Hospitalised sample)	113	Total prevalence of 39%		
[50] Ethiopia	To assess risk factors of acute respiratory infection among under five children attending public hospitals in Southern Tigray, Ethiopia	Case control study	Under five children (hospitalised sample)	288		Malnutrition, cow dung fuel use, presence of smoker in the family, low maternal literacy	
[51] Nigeria	To examine Indoor airborne microbial burden and risk of acute respiratory infections among children under five years	Case control study	Under-5 years (hospitalised sample)	132		Higher indoor bacterial counts	
[52] Nigeria	To evaluate housing quality and risk of acute respiratory infections among hospitalised children under five	Case control study	Hospitalised Under – 5 years (Hospitalised sample)	132		Damp roof, mould growths on walls and high indoor air relative humidity were risk factors	
[53] Ethiopia	To examine children under five from houses of unclean fuel sources and poorly ventilated houses have higher odds of suffering from acute respiratory infection	Case- control study	Children under – 5 years (hospitalised sample)	1144		Solid fuel for cooking, poor ventilated houses, large family size and carrying children whiles cooking	

## Findings

### Prevalence of ALRTIs in SSA

There is a high prevalence of ALRTIs among children under five in SSA. Thus, the prevalence rate of pneumonia includes 59.2% [46], 22.3% [15], 31.6% [14], 13.2% [18] and 9.0% [41]. Prevalence of Bronchiolitis reported in the studies were6.9% [14] and 1.9% [18]. Among reviewed studies, 60.2% [42] and 1.9% [18] were the highest and lowest reported prevalence of ALRTIs, respectively. Furthermore, seven studies reported a prevalence between 1 and 10% [14, 18, 22, 29, 30, 41, 44]. The majority of the reviewed studies that provided the rates were between 11 and 20% [18, 19, 28, 31-34, 47]. Additionally, seven studies reported a prevalence between 21 and 30% [7, 15-17, 23, 24, 35] and four studies recorded a prevalence between 31 and 40% [14, 36, 43, 49]. Only one study provided a prevalence between 41 and 50% [1]. Finally, three studies provided a prevalence between 51% and 57.5% and were among hospitalised samples [13, 46]. Compared to studies that used community samples, studies that used hospitalised samples reported relatively higher prevalence of ALRTIs. For instance, 6 studies that used hospitalised samples reported prevalence above 30% [1, 13, 14, 46, 49]. See details in Table 2.

### Risk factors of ALRTIs of children under five in SSA

The reviewed studies showed that children above the age of 2 years are at higher risk of ALRTIs [7, 13, 16, 19, 25, 33, 37, 43, 47]. However, this evidence is inconclusive because children under 2 years seem highly susceptible to ALRTIs compared to the rest of children under five [13, 16, 25]. Moreover, children under five of teenage mothers [16, 22, 25] and children from mothers above age 35 [26] are more susceptible to ALRTIs. Evidence strongly establishes that children of mothers with low education [14, 15, 22, 29, 31-33, 36, 48, 50] and lack of employment [26, 30] are more vulnerable to developing ALRTIs. However, evidence on the risk of children’s residence for ALRTIs is inconsistent as some studies attributed it to rural dwellings [16, 23, 43, 48] while others attributed it to urban areas [13, 14, 31]. The review further shows that high-order births [43], large family sizes (above five) or crowded households [14, 18, 19, 22, 28, 53] and low household income [43, 48] are likely to predispose children under five to ALRTIs. Finally, children under five in households with poor hygienic practices, such as poor or irregular handwashing [16, 38] and cockroach infestation [20] are likely to be diagnosed with ALRTIs.

Poor ventilation or the absence of windows [1, 26, 35, 53], households without separate kitchens [18] electricity [31], clean water or improved toilet facilities [33] put children at risk of ALRTIs. Also, evidence shows that inhouse cooking [28, 32, 34, 47], usage of solid fuel [20, 22, 27, 53] or unclean fuel such as cow dung [14, 22, 26, 43] increase the risk of children to ALRTIs, especially those living in households with inadequate ventilation. Perhaps, children under 5 years who are held by their mothers while cooking [26, 35] and exposure to unclean and solid fuels for cooking [20, 22, 27, 53] may be at risk of developing various ALRTIs. Moreover, children under five exposed to cigarette smoke [14, 15, 35, 50] and wood smoke [15] may suffer more from ALRTIs complications.

Malnourished children under five are more likely to suffer ALRTIs complications [14, 26, 28, 43, 44, 48, 50]. For instance, children under five who are underweight [22, 33] or stunted [33, 37], and those anaemic [13] and not exclusively breastfed or poorly breastfed [1, 14, 22, 36] are more likely to suffer ALRTIs. Unfortunately, unvaccinated children under five [1] or children who did not complete their vaccination [46] or did not receive vitamin A supplements [13, 29, 30] are at higher risk of ALRTIs. Perhaps, children with existing health issues such as HIV [15] and a history of diarrhoea [19, 29-31, 33] become more vulnerable to ALRTIs (See Table 3 for details).

**Table 3:** Risk factors of ALRTIs among children under five in SSA

Main theme	Risk factors	Authors
Malnutrition	Malnutrition	[14, 44, 28, 43, 13, 48, 50]
	Underweight	[22, 33]
	Stunting	[33, 26]
	Anaemia	[25]
	Lack of breastfeeding/exclusive breastfeeding	[1, 14, 22, 36]
	Children who did not receive vitamin A supplement	[29, 30, 25]
Household facilities	Poor ventilation/absence of windows	[1, 35, 48, 53]
	House without separate kitchen	[1, 18]
	Damp roof, mould growths on walls	[51]
	Households with unclean water	[51]
	Households with poor toilet facilities	[51]
	Living in house with no chimney, eaves space,	[34]
	Children in households with a low proportion of electricity	[31]
Lack of Immunisation	Failure to receive the second dose of the pentavalent vaccine	[46]
	Unvaccinated children	[1]
	Poor immunisation	[14]
Cooking	Inhouse cooking,	[32, 34, 47, 28]
	Unclean fuel/stove or biomas	[14, 22, 43, 48]
	Frequency of cooking	[34, 47]
	Children who were held by their mothers while cooking	[35, 48]
	Solid fuel for cooking,	[22, 53, 20, 27]
	Cow dung fuel use,	[34, 50]
Smoking	Cigarette smoking,	[15, 14, 35, 50]
	Exposure to wood smoke,	[15]
Socio demographic	Child born to women with no formal or primary education	[13-15, 22, 31, 31-33, 13, 50]
	Children > 2 years	[6, 33, 47, 19, 16, 43, 25, 37, 26]
	Children < 2 years	[16, 25, 37]
	Rural residency	[6, 16, 43, 13]
	Urban residency	[14, 31, 25]
	Mothers who do not listen to or watch television	[32]
	Younger maternal age < 20 years	[22, 16, 37]
	Age of the mother/ caregiver > 35 years	[48]
	Housewives/mothers with no occupation	[30, 48]
	High order birth	[43]
	Large family size (> 5)/crowded homes	[14, 18, 22, 19, 28, 53]
	Poor households	[43, 13]
Hygienic practices	No hand washing,	[16, 38]
	Cockroach infestation	[20]
Pre-existing conditions	History of diarrhoea	[31]
	HIV infection	[15]
Climate issues	Changing climate	[45]
	Higher indoor bacterial counts	[51]
	Exposure to high concentrations of PM_2.5_ is a risk factor	[21]
Pregnancy	Uterine irritability during pregnancy	[20]
	physical exercise during pregnancy	[20]
Chemical exposure	The use of agro-chemicals is a risk factor	[24]

### Interventions to reduce ALRTIs among children under five in SSA

Some reviewed studies reported interventions that have been put in place to protect children under five against ALRTIs. Evidence shows that health promotion strategies such as campaigns and health education have doubled the health-seeking behaviours of mothers of children under 5 years of age against ALRTIs in Uganda [43]. However, a report from Niger indicates that multiple home visits for post-hospitalisation health monitoring were not more effective at preventing ALRTIs-related morbidity and mortality among under five children [46]. Similarly, evidence from Ethiopia shows that improvements made to baking stoves had appreciable effect on ALRTI among under five children [47]. Furthermore, evidence shows misuse of antibiotics, anti-malaria and herbal medicines to treat ALRTIs among these children [41, 42].

## Discussions

Findings show a high prevalence (between 1.9% to 60.2%) of ALRTIs among children under five of age in Africa. Predictors of ALRTIs among under five children include poor education, poverty, malnutrition, exposure to second-hand smoke, poor ventilation, HIV, traditional cooking stoves, unclean fuel usage, poor sanitation facilities and unclean source of drinking water.

### Prevalence of ALRTIs

The prevalence of ALRTIs among children under 5 years in SSA is relatively higher, with most studies reporting a prevalence between 10 and 60%. Perhaps, the use of demographic health surveys and hospitalised samples in most studies might result in the high prevalence rate of ALRTIs among children.. For instance, high prevalence rate of ALRTIs may be found among hospitalised samples because mostly such population may present symptoms to hospitals for treatments or diagnosis. Pneumonia consistently showed a high prevalence among children under five in SSA, regardless of the study design [14, 18, 46]. Higher prevalence of pneumonia in children might be one of the contributors of high numbers of deaths reported in SSA due to pneumonia [3, 10]. Studies that relied on self-reported symptoms are likely to report misleading prevalence because self-report symptoms may not produce a good diagnosis of ALRTIs among children.

### Risk factors of ALRTIs among children under five in SSA

Evidence from reviewed studies shows that malnutrition affects children under five and is likely to expose these children to severe ALRTIs [26, 28]. Perhaps, ALRTIs are highly prevalent in SSA because of the increasing trend in malnutrition in SSA [54, 55]. Moreover, exclusive breastfeeding serves as a protective factor for children under five against ALRTIs [56]. Though almost all infants in SSA are breastfed, exclusive breastfeeding is less common [51]. This situation makes it difficult to protect children under five from ALRTIs because only 33% of infants are exclusively breastfed in SSA [51]. Unfortunately, the review found that children infected with HIV are highly vulnerable to ALRTIs [56, 57]. Thus, pneumonia, for instance, frequently occurs as an opportunistic infection in HIV-infected children, especially among children in a region highly burdened by HIV [45, 57].

Low maternal education and socioeconomic status and factors such as poor toilet and drinking water facilities are significantly associated with ALRTIs among children under five. The effect of socioeconomic and demographic determinants on morbidity and mortality among children is well-established [10]. However, factors such as the specific age of children under five, maternal age and residence settings as risk factors for ALRTIs among under five children in SSA have shown inconsistent results. These factors many need further research attention. Low socioeconomic status is likely to increase the ALRTIs risk for children under 5 years through several factors, such as poor nutritional status, poor housing conditions, overcrowding, the use of unclean fuel and reduced access to health care and preventive programmes [28].

Furthermore, children under five exposed to smoke through the use of hard and unclean fuels as well as second-hand tobacco smoke, are likely to suffer from ALRTIs. Although high-quality studies with robust designs are needed, this finding is relevant since almost 40% of the children globally, especially those in resource-poor settings like SSA, are exposed to second-hand tobacco smoke [10]. Furthermore, this finding is significant given the fact that most SSA countries are heavily reliant on unclean cooking fuels due to multifaceted socioeconomic difficulties.

### Interventions to reduce ALRTIs among children under five in SSA

There is a lack of evidence regarding existing interventions in improving ALRTIs among children under five in SSA. For instance, evidence shows that health promotion activities such as multiple home visits by community health nurses p are not effective in preventing ALRTIs among children under 5 years [46]. Perhaps, this finding may be due to a lack of high-quality studies exploring the benefits of home visits in preventing ALRTIs. In addition, evidence shows that using an improved cooking stove does not serve as a protective factor against ALRTIs among children under five in SSA [47]. Thus, this demonstrates the lack of intervention studies to guide policies. This provides an opportunity for designing intervention research for preventing ALRTIs from children under five in SSA.

### Policy recommendations

Education and socioeconomic development need to be included in the list of interventions to reduce ALRTI morbidities and mortalities among children under 5 years not only because it is their fundamental rights but also because multisectoral and interdisciplinary approaches that go beyond treatment are increasingly recognised as efficient ways to address global health inequalities and improving lives of our children. This review provides evidence to support the need for additional interventions with the greatest potential for reducing the burden of ALRTIs among under five children in SSA. There is a need for health systems in SSA to strengthen antenatal care, optimal maternal physical and mental health, and HIV control in HIV-infected mothers.

Additionally, good ventilation is to be encouraged in homes. Clean cooking fuels should be promoted through subsidised programmes and education. Policies should direct governments in SSA to invest in providing decent and affordable housing and sanitation facilities for its citizens. Primarily, quality housing should be provided to poor people and those in vulnerable situations, such as street mothers and displaced communities, to help protect their children from ALRTIs. In addition, intersectoral collaboration is essential to strengthen poverty alleviation strategies, improve living conditions, optimise child nutrition, ensure all children have access to clean running water, and reduce the burden of ALRTIs among under five children in SSA.

## Recommendations for future studies

High-quality studies such as randomised controlled-trials and longitudinal studies that adjust for confounding variables are needed to explore how health promotion strategies like the use of improved cooking stoves and nutritional interventions protect against ALRTIs among children under five in SSA. Furthermore, there is a need for high-quality studies to explore factors such as specific age and place of residence (Rural or urban) of children under five that make them more susceptible to ALRTIs. Though there is some evidence that second-hand tobacco smoke and mothers’ age contribute to ALRTIs, existing studies are insufficient to confirm these findings in SSA. Hence, there is a need for more studies to explore these linkages. Finally, studies evaluating existing interventions on reducing ALRTIs among under five children in SSA are needed to determine which type of interventions are most effective at reducing ALRTIs among this young population.

## Limitations

One major limitation of this review is its lack of accounting for confounders in most reviewed studies. Besides, studies that used self-report measures to estimate the prevalence of ALRTIs may impede the generalisation of findings. In addition, language limitations may prevent authors from retrieving studies published in other languages, which could have added more strength to this review. However, this review used a comprehensive search strategy to help map relevant evidence to inform policy, interventions and future research.

## Conclusion

This scoping review aims to map evidence on prevalence, risk factors and interventions of ALRTIs among children under 5 years in SSA to inform future studies and help develop robust and effective policies and interventions in preventing and protecting children under 5 years. The findings show a high prevalence of ALRTIs, especially pneumonia, among children under five in SSA. Poor education of caregivers, poverty, malnutrition, exposure to second-hand tobacco smoke, poor ventilation, HIV, traditional cooking stoves and unclean fuel usage, poor sanitation facilities and unclean source of drinking water makes children under five more vulnerable to ALRTIs in SSA. Intersectoral collaboration are essential in SSA to reduce the burden ALRTIs among children under five. There is a need to strengthen poverty alleviation strategies, improve living conditions, optimise child nutrition, and ensure that all children have access to clean running water on the continent.

## Data Availability

All data generated or analysed during this study are included in this published article.
